# Supplementary material for: Nucleation and Growth-Controlled Facile Fabrication of Gold Nanoporous Structures for Highly Sensitive Surface-Enhanced Raman Spectroscopy Applications
Source: Nanomaterials (Basel). 2021 Jun 1;11(6):1463. doi: 10.3390/nano11061463 (PMC8227128; doi:10.3390/nano11061463)
Supplement: Supplementary file 1 [file nanomaterials-11-01463-s001.zip › nanomaterials-1242013-supplementary.pdf]

nanomaterials

Article

Nucleation and Growth-Controlled Facile Fabrication of Gold Nanoporous Structures for Highly Sensitive Surface-Enhanced Raman Spectroscopy Applications

Eunji Lee and Sangwoo Ryu *

Department of Advanced Materials Engineering, Kyonggi University, Suwon 16227, Korea; eg29560@kyonggi.ac.kr
* Correspondence: sryu@kyonggi.ac.kr; Tel.: +82-31-249-9761

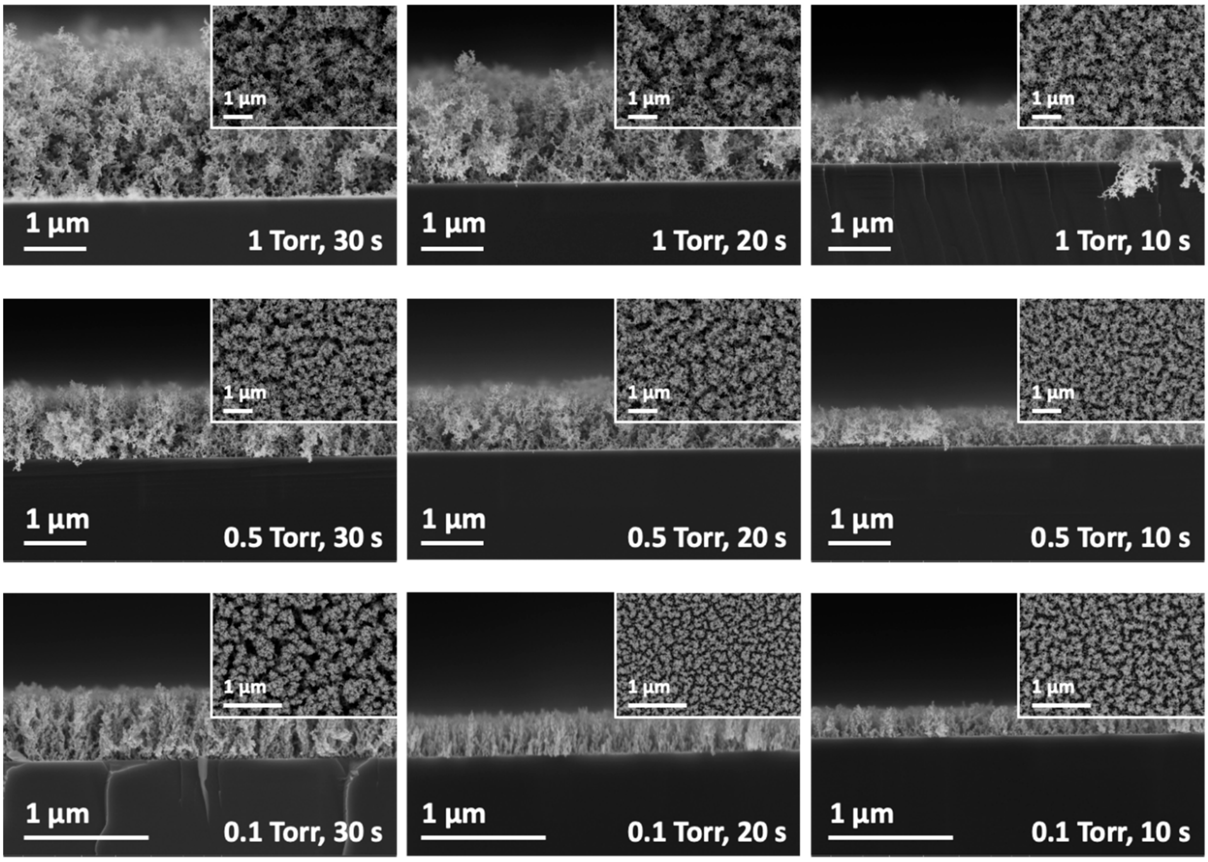

Figure S1. Various morphologies of porous Au synthesized by high-pressure thermal evaporation with different deposition pressure and time.

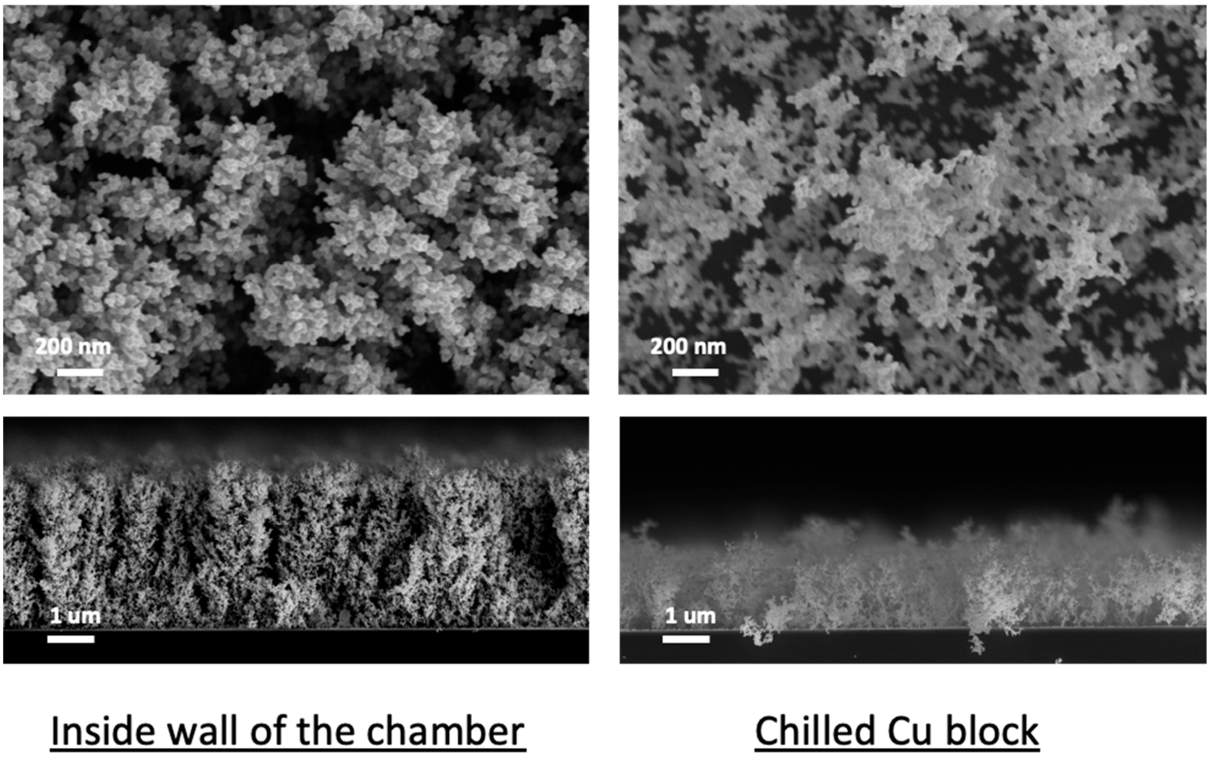

Figure S2. Different morphology of the porous Au nanostructures synthesized by high-pressure thermal evaporation depending on deposition temperature.
